# Supplementary material for: Effect of Dupilumab on Sexual Desire in Adult Patients with Moderate to Severe Atopic Dermatitis
Source: Medicina (Kaunas). 2022 Nov 23;58(12):1708. doi: 10.3390/medicina58121708 (PMC9782472; doi:10.3390/medicina58121708)
Supplement: Supplementary file 1 [file medicina-58-01708-s001.zip › medicina-2011620-supplementary.pdf]

Figure S1. Sexual Desire Inventory- 2 (SDI-2)[14-15] questionnaire administered to 328 patients with moderate-to-severe atopic dermatitis

1. During this last month, how often would you have liked to engage in sexual activity with a partner

(For example, touching each other's genitals, giving, or receiving oral stimulation, intercourse, etc.)?

- Not at all
- Once a month
- Once every two weeks
- Once a week
- Twice a week
- 3 to 4 times a week
- Once a day
- More than once a day

2. During this last month, how often have you had sexual thoughts involving a partner?

- 0) Not at all
- 1) Once a month
- 2) Once every two weeks
- 3) Once a week
- 4) Twice a week
- 5) 3 to 4 times a week
- 6) Once a day
- 7) More than once a day

3. When you have sexual thoughts how strong is your desire to engage in sexual behaviour with a partner?

No desire 0 1 2 3 4 5 6 7 8 Strong desire

4. When you first see an attractive person, how strong is your sexual desire?

No desire 0 1 2 3 4 5 6 7 8 Strong desire

5. When you spend time with an attractive person (for example, at work or school), how strong is your sexual desire?

No desire 0 1 2 3 4 5 6 7 8 Strong desire

6. When you are in romantic situations (such as a candle-lit dinner, a walk on the beach, etc.) how strong is your sexual desire?

No desire 0 1 2 3 4 5 6 7 8 Strong desire

7. How strong is your desire to engage in sexual activity with a partner?

No desire 0 1 2 3 4 5 6 7 8 Strong desire

8. How important is it for you to fulfil your sexual desire though activity with a partner?

Not at all important 0 1 2 3 4 5 6 7 8 Extremely important

9. Compared to other people of your age and sex, how would you rate your desire to behave sexually

with a partner?

Much less desire 0 1 2 3 4 5 6 7 8 Much more desire

10. During this last month, how often would you have liked to behave sexually by yourself (for example, masturbating, touching your genitals, etc.)?

- 0) Not at all
- 1) Once a month
- 2) Once every two weeks
- 3) Once a week
- 4) Twice a week
- 5) 3 to 4 times a week
- 6) Once a day
- 7) More than once a day

11. How strong is your desire to engage in sexual behaviour by yourself?

No desire 0 1 2 3 4 5 6 7 8 Strong desire

12. How important is it for you to fulfil your desires to behave sexually by yourself?

Not at all important 0 1 2 3 4 5 6 7 8 Extremely important

13. Compared to other people your age and sex, how would you rate your desire to behave sexually by yourself?

Much less desire 0 1 2 3 4 5 6 7 8 Much more desire

14. How long could you go comfortably without having sexual activity of some kind?

- Forever
- A year or two
- Several months
- A month
- Twice a week
- 3 to 4 times a week
- Once a day
- More than once a day
- A few weeks
- A week
- A few days
- One day
- Less than one day

If you have a score of 45 or lower you may be suffering from low desire and may be helped by contacting a practitioner who specializes in the medical diagnosis and treatment of sexual dysfunction
